# Closed genome sequence of *Fretibacterium fastidiosum*, a potential contributor to periodontal disease

**DOI:** 10.1128/mra.00004-25

**Published:** 2025-03-06

**Authors:** Asuka Mori, Mashu Kudoh, Masae Kuboniwa, Christian Rückert-Reed, Tobias Busche, Marion Eisenhut, Eiichiro Fukusaki, Andrea Bräutigam, Bianca Laker

**Affiliations:** 1 Department of Biotechnology, Graduate School of Engineering, Osaka University 594810 , Suita, Osaka, Japan; 2 Department of Preventive Dentistry, Graduate School of Dentistry, Osaka University 595046 , Suita, Osaka, Japan; 3 Center for Biotechnology (CeBiTec), Bielefeld University 9167 , Bielefeld, North Rhine-Westphalia, Germany; 4 Medical School OWL, Bielefeld University 9167 , Bielefeld, North Rhine-Westphalia, Germany; 5 Computational Biology, Faculty of Biology, Bielefeld University 98894 , Bielefeld, North Rhine-Westphalia, Germany; 6 Osaka University Shimadzu Omics Innovation Research Laboratories, Osaka University 13013 , Suita, Osaka, Japan; 7 Industrial Biotechnology Initiative Division, Institute for Open and Transdisciplinary Research Initiative, Osaka University 13013 , Suita, Osaka, Japan; Portland State University, Portland, Oregon, USA

**Keywords:** genomes, *Fusobacterium*, *Fretibacterium*, periodontal immunology

## Abstract

Human oral microbiome consists of diverse bacteria. Not all oral bacteria are well characterized due to challenges in cultivation *in vitro*. In this study, we report the closed genome sequence of one of the recently identified oral bacteria, *Fretibacterium fastidiosum*.

## ANNOUNCEMENT

Oral microbiome comprises more than 700 bacterial species (1). Over a third of these are yet uncultivated (2). *Fretibacterium fastidiosum*, previously uncultivated, requires stimulation by other oral bacteria for growth in broth culture and is possibly linked to periodontal disease (3–7). To further understand the association of this bacterium to periodontal disease, we here provide a contiguous genome sequence of *F. fastidiosum* SGP1. The existing reference strain genome, deposited under GenBank accession number GCA_000210715.1, consists of 362 contigs with 9.95% Ns.

*F. fastidiosum* SGP1 was obtained from the Japan Collection of Microorganisms (JCM:16858) and was cultivated using the same method proposed by Vartoukian et al. with some modifications (8). Briefly, *F. fastidiosum* was first cultured on blood agar plates cross-streaked with *Fusobacterium nucleatum* ATCC 25586 (from JCM:8532) to stimulate its growth. Colonies were transferred into Nutrient Broth no. 2 supplemented with 1% yeast extract, 0.1% l-cysteine hydrochloride hydrate, and filtered culture supernatants (50%, v/v) from broth cultures of *F. nucleatum*. Then, 2 × 10^9^ cells were collected by centrifugation. DNA was isolated using the Maxwell RSC Cultured Cells DNA Kit (Promega, Madison, Wisconsin, US) and Maxwell RSC Instrument (Promega). The DNA quality and quantity were assessed with the Tape Station (Agilent) and the Qubit fluorometer (Invitrogen). Sequencing libraries were prepared with the Ligation sequencing gDNA - Native Barcoding Kit 24 V14 (SQK-NBD114.24; Oxford Nanopore Technologies [ONT], Oxford, UK) following the manufacturer’s protocol. In the clean-up step, the long fragment buffer was used. One R10.4.1 flow cell was run for 19.5 h on a GridION (ONT). Bases were called using Dorado v7.2.13 with model dna_r10.4.1_e8.2_400bps_sup@v4.2.0 (ONT), producing 410,040 raw reads with an *N*_50_ of 6,365 bp. All programs were run with default parameters unless otherwise specified.

Adapter trimming was performed with cutadapt v4.8 setting “-e 0.2 --trimmed-only” with “-g AAGGTTAANNNNNNNNNNNNNNNNNNNNNNNNCAGCACCT” for 5′ adapter and “-a AGGTGCTGNNNNNNNNNNNNNNNNNNNNNNNNTTAACCTTAGCAAT” for 3′ adapter (9). A genome assembly was calculated with Flye v2.9.5 in assembly mode meta (10) and polished with racon v1.5.0 (11) and minimap v2.26 setting parameter “-ax map-ont” (12) and Medaka v2.0.1 setting “-m r1041_e82_400bps_sup_v4.2.0” (ONT). Overhangs were trimmed with Berokka v0.2.3 (https://github.com/tseemann/berokka), duplicated sequences were discarded with Circlator clean v1.5.5 (13), and sequences were adjusted to start at *dnaA* with Circlator fixstart. Phylogenetic classification was performed with the Type (Strain) Genome Server (TYGS) (14) and with BLASTN v2.16.1+ (WebBLAST) using the database core_nt (15, 16). Genome completeness was measured with benchmarking universal single-copy orthologs (BUSCO) v5.6.1 in mode geno (17). Genes were annotated with Prokka v1.14.6 (18), and dot plots were created with D-Genies v1.5.0 (19).

**Fig 1:**
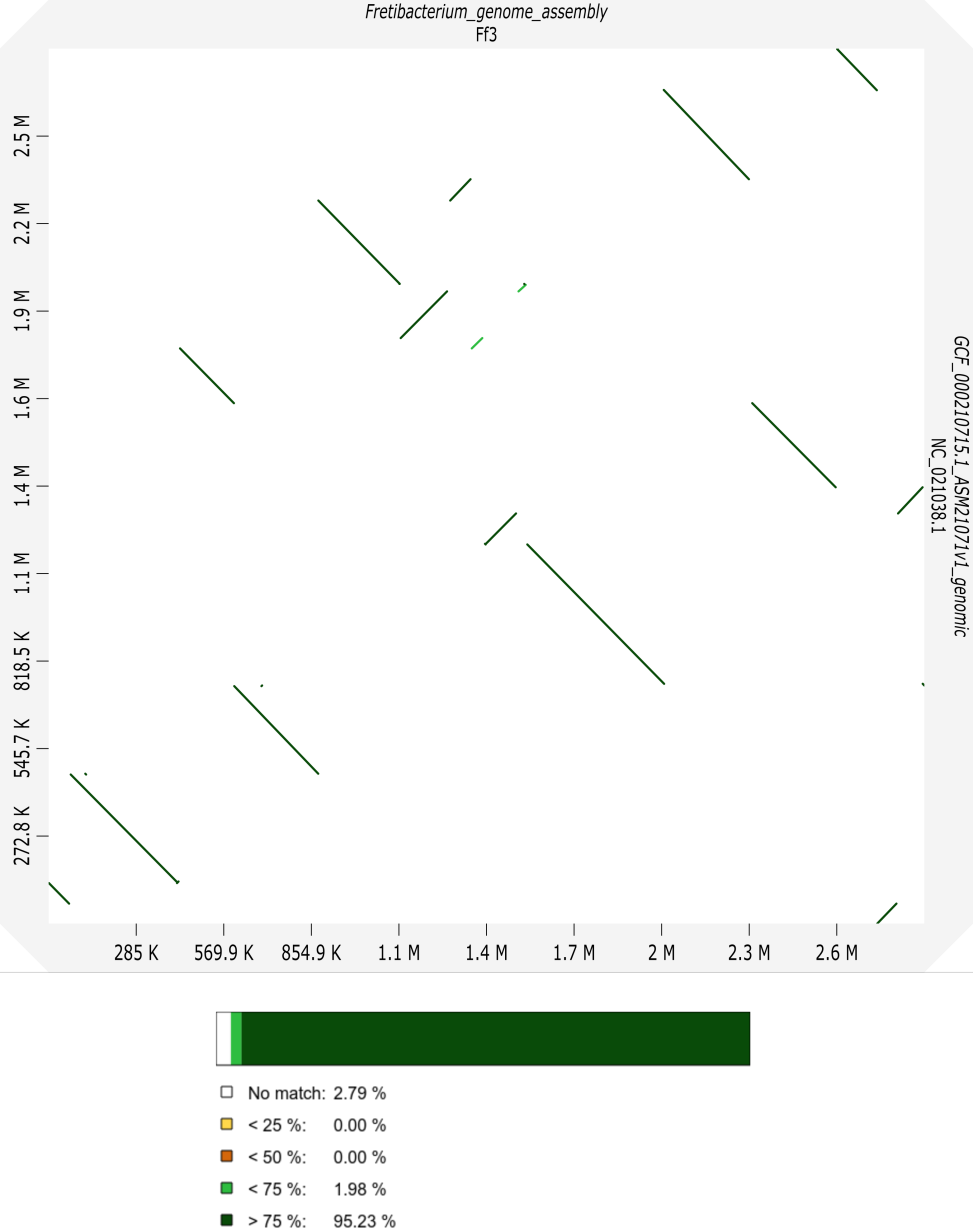
Sequence comparison between the reported circular genome contig of *F. fastidiosum* SGP1 (*x*-axis) and the gapped reference genome sequence of the same strain (GenBank accession number GCA_000210715.1; *y*-axis). The color code for the dot plot with alignment identities in percent is shown as a bar graph.

The genome of *F. fastidiosum* SGP1 and of *F. nucleatum* ATCC 25586 were both circularly assembled and have a high number of BUSCO genes for *Synergistota* (*F. fastidiosum*) or *Fusobacteriales* (*F. nucleatum*), classifying them as complete genome sequences. Relevant statistics are listed in Table 1. Figure 1 shows the comparison between the reported genome of *F. fastidiosum* SGP1 and the gapped reference genome sequence of the same strain (GenBank accession number GCA_000210715.1).

**TABLE 1:** Assembly statistics for the *F. fastidiosum* SGP1 and *F. nucleatum* ATCC 25586 genome sequences

Parameter	Finding for	
	*F. fastidiosum* SGP1	*F. nucleatum* ATCC 25586
Number of contigs	1	1
Length (bp)	2,849,698	2,166,339
GC content (%)	63.17	27.12
Genome coverage (x)	109	584
Total no. of genes	2,477	2,068
No. of protein-coding genes	2,418	2,005
No. of rRNAs	9	15
No. of tRNAs	49	47
No. of transfer messenger RNAs	1	1
BUSCO results (%)^*a*^		
Complete	82.3	99.2
Single copy	81.5	99.2
Duplicated	0.8	0.0
Fragmented	0.3	0.2
Missing	17.4	0.6

^*a*^ The databases used (and the number of searched BUSCOs) were as follows: *F. fastidiosum* SGP1, synergistetes_odb10 (655 BUSCOs); *F. nucleatum* ATCC 25586, fusobacteriales_odb10 (510 BUSCOs).

## Data Availability

The *F. fastidiosum* SGP1 and *F. nucleatum* ATCC 25586 genome sequences, corresponding gene annotations, and the raw read data are available in GenBank/ENA under BioProject accession number PRJEB82993. The GenBank/ENA accession number for the raw reads is ERR14050159. The GenBank/ENA accession number for the *F. fastidiosum* SGP1 assembly and annotation is GCA_964642095.1, and the number for the *F. nucleatum* ATCC 25586 assembly is GCA_964642115.1.
